# Psychological impacts of the COVID-19 pandemic on one-month postpartum mothers in a metropolitan area of Japan

**DOI:** 10.1186/s12884-021-04331-1

**Published:** 2021-12-28

**Authors:** Youji Takubo, Naohisa Tsujino, Yuri Aikawa, Kazuyo Fukiya, Momoko Iwai, Takashi Uchino, Megumu Ito, Yasuo Akiba, Masafumi Mizuno, Takahiro Nemoto

**Affiliations:** 1 Department of Neuropsychiatry, Toho University Graduate School of Medicine, 5-21-16 Omori-nishi, Ota-ku, Tokyo, 143-8540 Japan; 2 Department of Psychiatry, Saiseikai Yokohamashi Tobu Hospital, 3-6-1 Shimosueyoshi, Tsurumi-ku, Yokohama, Kanagawa 230-8765 Japan; 3 Department of Neuropsychiatry, Toho University Faculty of Medicine, 6-11-1 Omori-nishi, Ota-ku, Tokyo, 143-8541 Japan; 4 Department of Obstetrics and gynaecology, Saiseikai Yokohamashi Tobu Hospital, 3-6-1 Shimosueyoshi, Tsurumi-ku, Yokohama, Kanagawa 230-8765 Japan; 5 Tokyo Metropolitan Matsuzawa Hospital, 2-1-1 Kamikitazawa, Setagaya-ku, Tokyo, 156-0057 Japan

**Keywords:** Anxiety, COVID-19, Japan, Postnatal depression, Postpartum

## Abstract

**Background:**

The coronavirus disease 2019 (COVID-19) pandemic has recently become the most important issue in the world. Very few reports in Japan have examined the impact of the COVID-19 pandemic on peripartum mental health. We examined the status of postpartum mental health before and during COVID-19 pandemic from a consecutive database in a metropolitan area of Japan.

**Methods:**

The subjects were women who had completed a maternity health check-up at a core regional hospital in Yokohama during the period from April 1, 2017, to December 31, 2020. We collected the subjects’ scores for the Edinburgh Postnatal Depression Scale (EPDS) and the Mother-to-Infant Bonding Scale (MIBS) at 1 month postpartum. The subjects were divided into four groups (three Before COVID-19 groups and a During COVID-19 group). MANOVA and post-hoc tests were used to determine mental health changes in the postpartum period among the four groups.

**Results:**

The Before and During COVID-19 groups contained 2844 and 1095 mothers, respectively. There were no significant difference in the total scores of the EPDS and MIBS among the four groups. However, the EPDS items related to anxiety factors were significantly higher and the EPDS items related to anhedonia and depression factors (excluding thoughts of self-harm) were significantly lower in the During COVID-19 group.

**Conclusion:**

The EPDS scores changed in connection with the COVID-19 pandemic. Anxiety, which represent hypervigilance, was significantly higher and anhedonia and depression were significantly lower in the During COVID-19 group. Our results may reflect COVID-19-related health concerns and a lack of social support caused by the COVID-19 pandemic.

## Background

The coronavirus disease 2019 (COVID-19) pandemic has recently become the most important issue in the world, and no one has been unaffected by its impact [1]. Fear and worry about COVID-19, the impact of the state of emergency on daily life, and the difficulty in predicting the situation have caused much stress and have led to mental health problems. Evidence of COVID-19 and its relation to mental health issues has been published in different countries, and the psychological impact of COVID-19 is already obvious, both in the general population and in people with existing mental disorders [2, 3]. Previous studies have shown that the COVID-19 pandemic has caused a sharp increase in the prevalence of anxiety and depressive disorders among the general adult population in the world [4–8]. In particular, people aged 18 to 34 years, women, and people living with infants are more vulnerable to COVID-19-related stress [2, 9]. These studies suggest that young women who live with infants may be particularly susceptible to mental distress during the COVID-19 pandemic.

Mental illnesses, such as depressive and anxiety disorders, are among the most common morbidities during pregnancy and in the postpartum period, and the existence of mental distress in mothers can have adverse effects on the psychological development of their children [10–13]. Concern over COVID-19 causing critical illness or death is at the core of everyone’s anxiety. In addition, mothers may be worried more about their children than themselves. The impact of the COVID-19 pandemic on the mental health of perinatal mothers has also been clarified [14, 15]. Some studies have demonstrated high rates of stress, anxiety, and depressive symptoms, including thoughts of self-harm, in pregnant and postpartum mothers around the world during the COVID-19 pandemic [16–23]. A systematic review and meta-analysis of eight studies using the Edinburgh Postnatal Depression Scale (EPDS) and the State-Trait Anxiety Inventory (STAI) examined the impact of the COVID-19 pandemic on anxiety and depression in pregnancy and the perinatal period [22]. In that study, the effect sizes and standardized mean differences (SMDs) and the corresponding 95% confidence intervals (CIs) were calculated using the random-effects model. Whereas the EPDS score did not reach a statistically significant difference, the STAI score was significantly higher during the pandemic than in previous non-pandemic times [22]. In addition, a study of 2740 pregnant women during the pandemic found that stopping face-to-face prenatal visits and modifications to birth plans were strongly associated with anxiety [20]. Similarly, previous studies have suggested that social isolation and quarantine exacerbated depression and anxiety among pregnant women [17, 24].

Regarding mother-to-infant bonding, maternal bonding is the creation of an emotional bond between a mother and her new-born baby, and bonding failure is characterized by an aversion to the infant and a marked impairment in interactions, including decreased maternal affection, increased irritability, and rejection of the infant [25]. Impaired bonding has been suggested to lead to maltreatment and child abuse [25]. The coexistence of bonding failure and postpartum depression has been reported, although a causal relationship remains unclear [26, 27]. Very few studies have evaluated the impact of the COVID-19 pandemic on mother-to-infant bonding [28].

The worldwide COVID-19 pandemic is ongoing in Japan, especially in the Tokyo metropolitan area. However, very few reports have examined the impacts of the COVID-19 pandemic on peripartum depressive and anxious symptoms and mother-to-infant bonding in Japan [29, 30]. While an online EPDS-based survey of pregnant women during the COVID-19 pandemic was conducted in Japan, the respondents were not compared with a control group that reflected the situation before the COVID-19 pandemic [30]. The total number of people infected with COVID-19 in Kanagawa Prefecture, which is part of the Tokyo metropolitan area, was 61,516 at the end of May 2021 [31]. Kanagawa is located next to Tokyo, which is the area of Japan with the highest number of total COVID-19 cases. To the best of our knowledge, no data on the impact of the COVID-19 pandemic on mental health in postpartum women has been reported in Japan to date; specifically, no comparisons with a control group that reflects the situation before the COVID-19 pandemic have been made. To provide integrated postpartum treatment during the COVID-19 era, an examination of the current status of postpartum mental health, such as depressive and anxious symptoms as well as mother-to-infant bonding, is needed. Accordingly, the aim of the present study was to investigate the status of postpartum mothers’ mental health taking into consideration the influence of the COVID-19 pandemic in a metropolitan area of Japan by comparing the EPDS and Mother to Infant Bonding Scale (MIBS) scores of mothers before and during the COVID-19 pandemic.

## Methods

### Procedures and subjects

This study consisted of a retrospective chart review that was aimed at examining the influence of the COVID-19 pandemic on women’s mental health at 1 month postpartum. We used a naturalistic observation methodology performed in a clinical setting, and a control group was not utilized. The Saiseikai Yokohamashi Tobu Hospital is a regional core general hospital that covers the eastern area of Yokohama City in Kanagawa Prefecture; it also accepts people with COVID-19. For the early detection and intervention of postpartum mental health problems and the identification of their causes, all women are asked to complete the Japanese versions of the EPDS and MIBS at the time of a one-month maternity health check-up performed by midwives, and a continuous hospital database of this information has been maintained at the Department of Psychiatry since April 1, 2017. The subjects were women who gave birth and had a maternity health check-up at 1 month after childbirth in the Obstetrics and Gynaecology Departments of the Saiseikai Yokohamashi Tobu Hospital. We set the investigation period as lasting from April 1, 2017, to December 31, 2020.

The study protocol was approved by the Ethics Committees of Saiseikai Yokohamashi Tobu Hospital (20200129). We obtained informed consent in the form of an opt-out on a hospital website after obtaining procedure approval (December 2020). We set the opt-out period for 3 months after answering the questionnaires, and no one declined to participate in this study. Therefore, all the participants’ administration had been completed by March 2021. The EPDS and MIBS scores at 1 month postpartum and the subjects’ demographic and obstetric information, including the weeks of childbirth, delivery style, parity, and birth weight of the child, were collected by researchers Y.T., Y.Ai., and K.F. from the continuous database and medical charts.

The primary outcome consisted of the EPDS and MIBS scores. Cox et al. developed the EPDS, and the Japanese version was reported by Okano to have a good internal consistency [32, 33]. Taylor et al. developed the MIBS, and the Japanese version was translated and validated by Yoshida [34, 35]. Both the EPDS and the MIBS are self-reported instruments with 10 items rated on a 4-point scale with total scores ranging from 0 to 30 [32–35]. The higher the score of EPDS and MIBS, the worse the symptoms of postpartum depression and mother-to-infant bonding, respectively. In the Japanese version of the EPDS, the optimal cut-off score was 8/9 for screening for perinatal depression [33]. A recent Japanese study using a large dataset revealed a three-factor structure model consisting of “Anhedonia” (Items 1 and 2), “Anxiety” (Items 3, 4, 5, and 6), and “Depression” (Items 7, 9, and 10). This model demonstrated a high goodness-of-fit [36]. On the other hand, a Japanese study using a cluster analysis suggested the presence of mothers with pathological maternal bonding (14.4%), with an optimal MIBS cut-off score of 4/5 at 1 month after delivery [37]. The MIBS was suggested to have a two-factor structure composed of “Lack of affection” (Items 1, 6, 8, and 10) and “Anger and rejection” (Items 2, 3, 5, and 7) [35]. We used the above cut-off scores and factor structures in the present analysis.

This study was performed as part of the Mental health and Early Intervention in the Community-based Integrated care System (MEICIS) project, which is supported by a Health Labour Sciences Research Grant (19GC1015). The study was performed in accordance with the latest version of the Declaration of Helsinki (October 2013).

### Statistical analysis

**Fig. 1:**
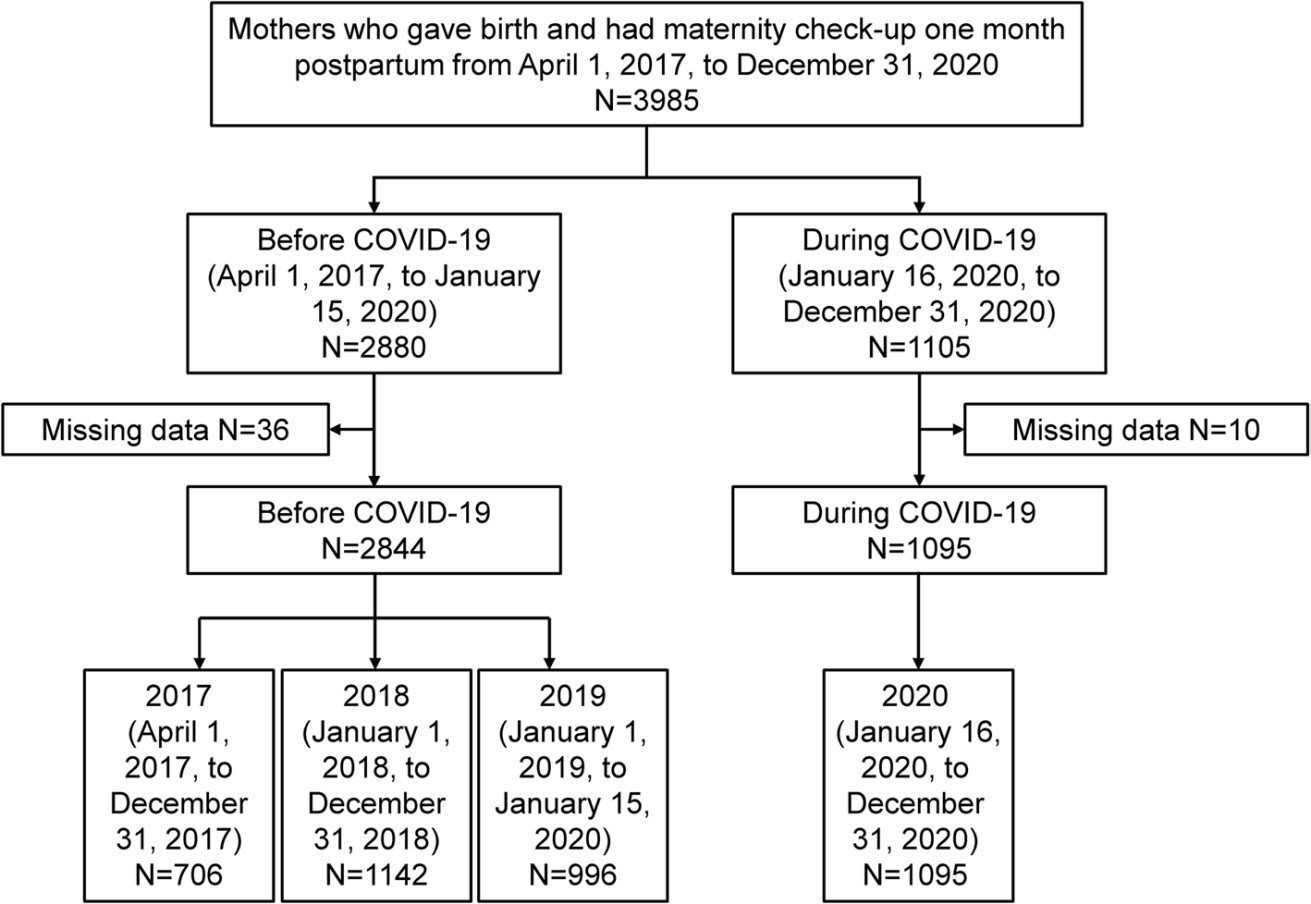
Study flow chart

A total of 3985 women delivered during the period, and only 46 women did not complete the EPDS or the MIBS questionnaire. The proportion of women excluded in the analysis was small (1.2%). The subjects were divided into two groups based on a cut-off date of January 16, 2020, when the first case of COVID-19 was reported in Japan. Subjects who answered the questionnaires during the period from April 1, 2017, to January 15, 2020, were included in the “Before COVID-19” group, while those who answered the questionnaires during the period from January 16, 2020, to December 31, 2020, were included in the “During COVID-19” group. Then, we divided the Before COVID-19 group into three subgroups according to year (2017: April 1, 2017, to December 31, 2017; 2018: January 1, 2018, to December 31, 2018; 2019: January 1, 2019, to January 15, 2020). To examine the impact of the COVID-19 pandemic more precisely, we then compared each primary outcome among the four groups (the three Before COVID-19 subgroups and the During COVID-19 group [2020: January 16, 2020, to December 31, 2020]). The study flow chart is shown in Fig. 1. We used a multivariate analysis of variance (MANOVA) followed by post-hoc Tukey’s honestly significant difference (HSD) and chi-square tests to compare demographic and clinical variables (such as age, parity, and delivery style; and the total scores of EPDS and MIBS) between the groups. The Kruskal-Wallis test was used to test for the proportion of postpartum depression or postpartum pathological maternal bonding among the groups. All the statistical analyses were conducted using IBM SPSS, version 26.0.

## Results

The mean age of all the participants was 33.4 (SD = 5.1) years. The proportions of participants who were over 35 years old (late childbearing), were primipara, had a full-term birth (> 38 weeks), delivered vaginally, and had a singleton live birth were 42.1, 48.5, 95.9, 71.8, and 97.9%, respectively. Regarding the birth weight of the infant, 88.3% of the infants were of normal weight. None of the mothers in this study contracted COVID-19 during pregnancy or the postpartum period. The Before COVID-19 group was also divided into three subgroups according to year (2017: *n* = 706; 2018: *n* = 1142; 2019: *n* = 996). We compared the average EPDS and MIBS scores and general and obstetric characteristics for the three Before subgroups plus the During COVID-19 group (2020: *n* = 1095). The general and obstetric characteristics of the participants are shown in Table 1. A one-way Analysis of variance (one-way ANOVA) and the chi-square test showed no significant difference in general and obstetric characteristics between the Before COVID-19 and the During COVID-19 groups.

**Table 1:** General and obstetric characteristics of subjects before and during the COVID-19 pandemic

	Before COVID-19 group						During COVID-19 group		*P* ^a^ value
	2017		2018		2019		2020		
	*n* = 706		*n* = 1142		*n* = 996		*n* = 1095		
	n	%	n	%	n	%	n	%	
Age									
Mean (SD)	33.7	5.2	33.4	5.0	33.2	5.1	33.2	5.2	0.178^b^
< 34	395	55.9%	663	58.1%	578	58.0%	645	58.9%	0.665
≧35	311	44.1%	479	41.9%	418	42.0%	450	41.1%	
Parity									
Primipara	331	46.9%	565	49.5%	494	49.6%	520	47.5%	0.552
Multipara	375	53.1%	577	50.5%	502	50.4%	575	52.5%	
Birth weeks									
Premature delivery (< 37 weeks)	34	4.8%	43	3.8%	42	4.2%	44	4.0%	0.736
Full term birth (> 38 weeks)	672	95.2%	1099	96.2%	954	95.8%	1051	96.0%	
Delivery style									
Vaginal delivery	501	71.0%	810	70.9%	723	72.6%	793	72.4%	0.756
Caesarean Section	205	29.0%	332	29.1%	273	27.4%	302	27.6%	
Birth									
Single birth	690	97.7%	1117	97.8%	948	95.2%	1073	98.0%	0.719
Multiple birth	16	2.3%	25	2.2%	18	1.8%	22	2.0%	
Birth weight of infant									
Normal or high birth weight infant (> 2500 g)	643	89.1%	1015	87.0%	902	89.0%	989	88.5%	0.413
Low birth weight infant (< 2500 g)	79	10.9%	152	13.0%	112	11.0%	128	11.5%	

^a^Analysed by the chi-square test; ^b^ one-way Analysis of variance

Comparisons of the EPDS and MIBS scores for each item using the MANOVA are shown in Table 2. The analysis revealed a significant overall difference between the four groups (Wilks’ lambda = 0.379, *F* (60, 11,684) = 74.6, *p* < 0.001) for the primary outcomes.

**Table 2:** Comparison of EPDS and MIBS scores for each item

												Post-hoc (Tukey’s HSD)								
	2017		2018		2019		2020		MANOVA			2017 vs 2018	2017 vs 2019	2017 vs 2020		2018 vs 2019	2018 vs 2020		2019 vs 2020	
	*n* = 706		*n* = 1142		*n* = 996		*n* = 1095													
	Mean	SD	Mean	SD	Mean	SD	Mean	SD	F	*p*		*p*	*p*	*p*		*p*	*p*		*p*	
EPDS1	0.98	0.87	0.96	0.88	0.93	0.89	0.07	0.29	351.607	0.000	**	0.961	0.590	0.000	**	0.813	0.000	**	0.000	**
EPDS2	0.45	0.73	0.41	0.70	0.45	0.74	0.16	0.44	48.756	0.000	**	0.501	1.000	0.000	**	0.352	0.000	**	0.000	**
EPDS3	0.24	0.52	0.22	0.52	0.27	0.57	0.95	0.88	312.737	0.000	**	0.905	0.758	0.000	**	0.242	0.000	**	0.000	**
EPDS4	0.24	0.48	0.20	0.46	0.24	0.48	0.86	0.91	271.466	0.000	**	0.747	1.000	0.000	**	0.642	0.000	**	0.000	**
EPDS5	0.09	0.31	0.09	0.34	0.09	0.34	0.46	0.75	149.236	0.000	**	0.985	0.994	0.000	**	1.000	0.000	**	0.000	**
EPDS6	0.12	0.36	0.13	0.42	0.18	0.49	1.13	0.80	812.361	0.000	**	0.928	0.128	0.000	**	0.273	0.000	**	0.000	**
EPDS7	0.92	0.94	0.87	0.91	0.87	0.91	0.26	0.58	145.689	0.000	**	0.576	0.665	0.000	**	0.999	0.000	**	0.000	**
EPDS8	1.16	0.82	1.19	0.79	1.17	0.82	0.50	0.70	196.613	0.000	**	0.871	1.000	0.000	**	0.889	0.000	**	0.000	**
EPDS9	0.47	0.65	0.46	0.67	0.44	0.66	0.24	0.53	32.159	0.000	**	0.997	0.842	0.000	**	0.892	0.000	**	0.000	**
EPDS10	0.07	0.29	0.06	0.28	0.07	0.30	0.08	0.34	1.112	0.343										
MIBS1	0.14	0.38	0.15	0.41	0.12	0.36	0.12	0.36	2.048	0.105										
MIBS2	0.56	0.67	0.56	0.72	0.58	0.71	0.55	0.71	0.304	0.822										
MIBS3	0.18	0.41	0.17	0.40	0.18	0.42	0.18	0.41	0.080	0.971										
MIBS4	0.05	0.28	0.04	0.25	0.04	0.23	0.05	0.31	0.748	0.523										
MIBS5	0.09	0.30	0.10	0.33	0.11	0.33	0.10	0.30	0.307	0.820										
MIBS6	0.58	0.73	0.50	0.68	0.57	0.73	0.48	0.67	4.562	0.003	*	0.080	0.992	0.023	*	0.095	0.948		0.025	*
MIBS7	0.07	0.32	0.06	0.30	0.07	0.33	0.05	0.28	0.795	0.497										
MIBS8	0.10	0.36	0.09	0.37	0.08	0.34	0.09	0.35	0.321	0.810										
MIBS9	0.06	0.25	0.06	0.30	0.08	0.35	0.06	0.31	1.273	0.282										
MIBS10	0.21	0.50	0.20	0.49	0.20	0.52	0.17	0.48	1.510	0.210										

* *p* < 0.05, ** *p* < 0.001

The average EPDS scores for each item in the 2017, 2018 and 2019 subgroups were statistically consistent, and only those for 2020 differed significantly (except for EPDS Item 10). The scores for Items 3, 4, 5 and 6 were significantly higher during COVID-19, while the scores for Items 1, 2, 7, 8 and 9 were significantly lower during COVID-19. Regarding the factor structure, the sum score for “EPDS anxiety (Items 3, 4, 5 and 6)” [*F* = 643.728, *p* < 0.001, η^2^ = 0.329] was significantly higher during COVID-19, while the sum scores for “EPDS anhedonia (Items 1, and 2)” [*F* = 249.236, *p* < 0.001, η^2^ = 0.160] and “EPDS depression (Items 7, 9 and 10)” [*F* = 95.415, *p* < 0.001, η^2^ = 0.068] were significantly lower during COVID-19.

**Fig. 2:**
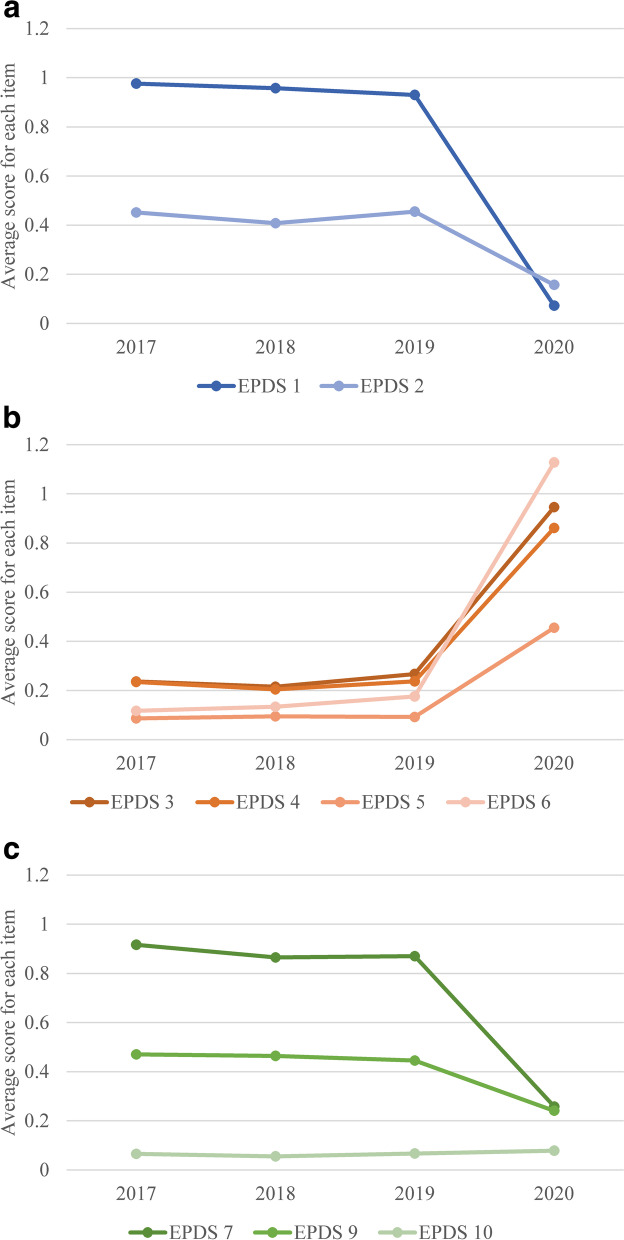
**a** Comparison of average scores by year (EPDS, Anhedonia items). **b** Comparison of average scores by year (EPDS, Anxiety items). **c** Comparison of average scores by year (EPDS, Depression items). Note: EPDS: Edinburgh Postnatal Depression Scale; 2017: April 1, 2017, to December 31, 2017 (*n* = 706); 2018: January 1, 2018 to December 31, 2018 (*n* = 1142); 2019: January 1, 2019 to January 15, 2020 (*n* = 996); 2020: January 16, 2020, to December 31, 2020 (*n* = 1095)

**Fig. 3:**
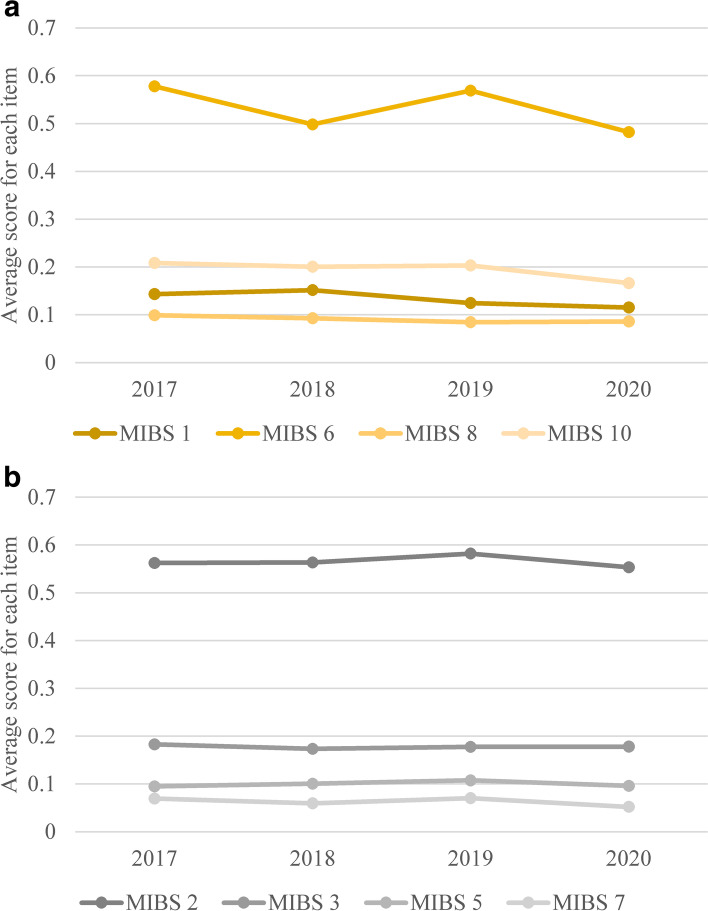
**a** Comparison of average scores by year (MIBS, Lack of affection items). **b** Comparison of average scores by year (MIBS, Anger and rejection items). Note: MIBS: Mother-to-Infant Bonding Scale; 2017: April 1, 2017, to December 31, 2017 (*n* = 706); 2018: January 1, 2018 to December 31, 2018 (*n* = 1142); 2019: January 1, 2019 to January 15, 2020 (*n* = 996); 2020: January 16, 2020, to December 31, 2020 (*n* = 1095)

On the other hand, the score for MIBS Item 6 showed significant difference between the four groups, whereas no significant differences in the other MIBS items were seen. Post hoc comparison for the score for MIBS Item 6 yielded significant difference in 2017 vs. 2020 (*p* = 0.023), and 2019 vs. 2020 (*p* = 0.025), however there were no significant difference in 2018 vs. 2020 (*p* = 0.948). Comparisons of each item in the factor structure are shown in Fig. 2a (EPDS, Anhedonia items), 2b (EPDS Anxiety items), 2c (EPDS, Depression items), 3a (MIBS, Lack of affection items) and 3b (MIBS, Anger and rejection items) (Fig. 3).

No significant difference in the average total scores of the EPDS (2017: 4.72; 2018: 4.59; 2019: 4.71; and 2020: 4.70; *F* = 0.247, *p* = 0.864, η^2^ = < 0.001) or the MIBS (2017: 2.04; 2018: 1.94; 2019: 1.84; and 2020: 1.84; *F* = 1.587 *p* = 0.190, η^2^ = 0.001) was seen, using a one-way ANOVA.

Regarding the cut-off score, the Kruskal-Wallis test showed no significant differences in the proportion of postpartum depression and postpartum pathological maternal bonding among the three Before COVID-19 groups and the During COVID-19 group (postpartum depression; 2017: 17.0%; 2018: 14.2%; 2019: 15.3%; and 2020: 15.8%; χ^2^ = 2.800, *p* = 0.423; postpartum pathological maternal bonding; 2017: 12.5%; 2018: 12.9%; 2019: 12.4%; and 2020: 10.7%; χ^2^ = 2.906, *p* = 0.406).

**Fig. 4:**
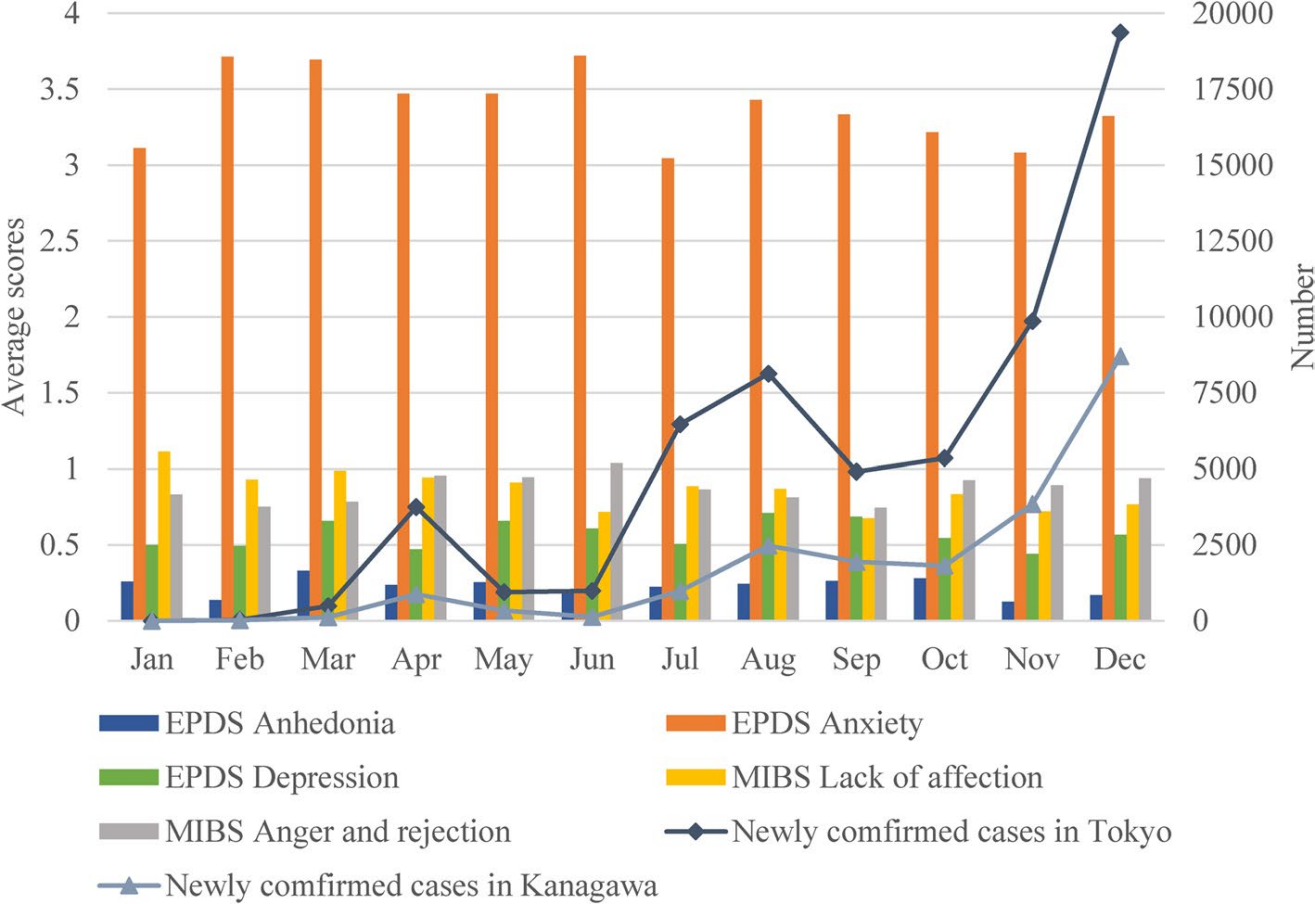
The newly confirmed cases of COVID-19 in Tokyo and Kanagawa and the average sum scores for each factor of the EPDS and MIBS during twelve time periods from January 16, 2020, to December 31, 2020. Surveillance data of COVID-19 were obtained from the official website of the Japanese Ministry of Health, Labour and Welfare (https://covid19.mhlw.go.jp/en/) [38]. Note: EPDS: Edinburgh Postnatal Depression Scale; MIBS: Mother-to-Infant Bonding Scale; EPDS Anhedonia: the sum of the EPDS scores for Items 1 and 2; EPDS Anxiety: the sum of the EPDS scores for Items 3, 4, 5, and 6 scores; EPDS Depression: the sum of the EPDS scores for Items 7, 9, and 10; MIBS Lack of affection: the sum of the MIBS scores for Items 1, 6, 8, and 10; MIBS Anger and rejection: the sum of the MIBS scores for Items 2, 3, 5, and 7

Furthermore, we also divided the During COVID-19 group into twelve subgroups according to month. To clarify the changes in scores during the COVID-19 pandemic and the relationships with the outbreak course, we compared the average sum scores for each factor of the EPDS and MIBS among these twelve subgroups with the number of the newly confirmed cases of COVID-19 in Tokyo and Kanagawa (Fig. 4) [38].

No significant changes in the sum score of each factor of the EPDS and MIBS were seen among these periods (EPDS anhedonia, *F* = 0.726, *p* = 0.714, η^2^ = 0.007; EPDS anxiety, *F* = 0.838, *p* = 0.602, η^2^ = 0.008; EPDS depression, *F* = 0.616, *p* = 0.816, η^2^ = 0.006; MIBS lack of affection, *F* = 0.640, *p* = 0.795, η^2^ = 0.006; MIBS anger and rejection, *F* = 0.625, *p* = 0.809, η^2^ = 0.006), using the one-way ANOVA. With reference to Fig. 4, the monthly changes of the sum score for each factor and the number of newly confirmed cases of COVID-19 seemed to be poorly related.

## Discussion

### COVID-19 as a disaster

The perspective of disaster mental health may be pivotal to interpreting the results of this study. Although an established definition of ‘disaster’ does not exist, disasters have been said to have three important characteristics. The first is a threat of harm or death to many people. The second is a disruption in social processes, services, and networks. And the third is an effect on mental and physical outcomes. The COVID-19 pandemic can be defined as a worldwide disaster [39]. Exposure to disasters has been associated with various mental health problems. Reportedly, women are less likely to be resilient during the post-disaster period than men, regardless of the type of disaster [40]. People living with children are also less likely to be resilient, perhaps because of their concerns and responsibilities towards their children. Longitudinal studies have shown that post-disaster mental health problems generally peak within a year and then improve, but in some people the symptoms persist [41]. However, COVID-19 is an ongoing disaster with no sign of a worldwide end. Therefore, mental health problems may become more serious in the future. The present study may serve as an important resource for clarifying the longitudinal course of postpartum maternal mental health during the COVID-19 pandemic.

### Higher levels of anxiety during COVID-19

Our survey revealed that during the COVID-19 pandemic, new mothers had higher levels of anxiety than those who gave birth before COVID-19. Pregnancy and childbirth can be stressful times for women even during normal circumstances; factors related to the COVID-19 pandemic may have further increased pregnancy- and childbirth-related anxiety. Previous articles have suggested that many people were worried about transmitting COVID-19 to their families, and a certain number of people exhibited pathological health anxiety characterised by an excessive fear of COVID-19 [7, 42]. Furthermore, a recent study revealed that COVID-19-related health worries may exacerbate mental health problems among pregnant women [43]. In addition, the unpredictability and uncertainty of the COVID-19 pandemic may also increase anxiety. Given the high degree of transmissibility and potential lethality of COVID-19, we believe that anxiety levels increased because of concerns over transmitting COVID-19 to their new-born child and cognitive changes arising from alertness to potential threats.

Furthermore, COVID-19-related physical distancing might have led to social isolation, limited access to basic services, and decreased family and social support [26, 29]. In Japan, many women have been greatly influenced by the COVID-19 pandemic during the course of their pregnancy and postpartum period, including changes in the place of delivery (8%), the cancellation of planned formal or informal support (23%) and the cancellation of parenting classes (79%) [29, 30]. Our investigation might reflect COVID-19-related health worries and a lack of social support because of the COVID-19 pandemic. The increase in anxiety among peripartum women during the COVID-19 pandemic was consistent with the results of a previous meta-analysis [22]. Anxiety during the pregnancy period can reportedly have negative effects on a child’s mental development, internalising problems, and cognitive function [44, 45]. Therefore, the current anxiety of perinatal women must be better understood, and an integrated approach to providing care is needed.

### Relationships among anxiety, anhedonia, and depression

Our results showed that depression and anhedonia were lower during the COVID-19 pandemic, and this result seems to be inconsistent with previous studies [22, 23]. However, hypervigilance might be a key to interpreting these results [39, 40]. Hypervigilance is a physiological and cognitive state of persistent hyperarousal and alertness against potential threats that allows a threat to be easily detected and a quick response to be made in potentially dangerous situations [46]. Hypervigilance might be the opposite states of depression and anhedonia. If anxiety and stress regarding the threat posed by COVID-19 cause hypervigilance, then the relative reduction in depression and anhedonia might also reflect the influence of hypervigilance [46]. Therefore, lower levels of anhedonia and depression might not necessarily mean that the pandemic has had a positive impact. In the present study, the results were considered to be consistent with mental health during a disaster. The EPDS results appear to be largely interpretable based on the concept of hypervigilance with the possible exception of Item 7, which may be slightly inconsistent. Item 7 consists of insomnia, which should be exacerbated during the COVID-19 pandemic because insomnia is one of the characteristics of hypervigilance [46]. Other factors may have an impact on the improvement of apparent insomnia.

When hypervigilance becomes a chronic condition characterised by sustained activation and failure to deregulate warning responses, people may experience impairments in their quality of life, such as the exacerbation of depression and anhedonia. A previous longitudinal study using path analysis attempted to clarify the relationship among anhedonia, anxiety and depression, and the results suggested that anxiety led to anhedonia and then to depression over time [47]. This study suggests that the chronic impact of the COVID-19 pandemic may lead to an eventual reverse in the downward trend of anhedonia and depression. The outcome will depend on multiple factors and the presence of resilience as a normal coping and adaption; thus, the changes associated with chronic courses should be followed continuously [48].

Of note, suicidal ideation (EPDS item 10), which is a component of the “EPDS Depression” factor, did not differ significantly between the Before and During COVID-19 groups, although the total depressive symptoms decreased during COVID-19. The suicide rate of peripartum women in Japan is estimated to be 8.7 per births of 100,000, which is higher than that in Western countries [49]. Furthermore, violent methods of suicide attempts are associated with critical perinatal outcomes [50]. Economic issues associated with the COVID-19 pandemic may also contribute to the exacerbation of suicidal ideation. Suicide ideation must be carefully considered because suicide attempts can lead to fatal consequences for both mother and child.

### Above cut-off

The prevalence of postpartum depression in Western countries is estimated to be 13–19% [51]. In Japan, a recent meta-analysis involving one hundred thousand Japanese women found that the prevalence of postpartum depression at one month after childbirth was 14.3% [52]. No significant difference in the prevalence of postpartum depression was seen between the three Before and the During COVID-19 groups, and the prevalence was almost equal to that reported in a recent Japanese meta-analysis [52]. As of December 2020, our results suggested that COVID-19 had little effect on the prevalence of postpartum depression and pathological maternal bonding.

### Maternal bonding

MANOVA revealed only the score for MIBS Items 6 was significant difference between the four groups, however, post hoc comparison showed there were no significant difference in 2018 vs. 2020. Therefore, this difference does not seem to be important clinically. Previous studies have suggested that anxiety was negatively associated with mother-to-infant bonding and that depressive symptoms were predictors of future bonding [53, 54]. Since qualitative changes in the EPDS subscales have occurred, maternal bonding may change in the future. Moreover, as the COVID-19 pandemic becomes increasingly chronic, it may come to have a negative influence on maternal bonding.

### Changes in mean scores of EPDS and MIBS throughout the COVID-19 pandemic

Figure 4 show the changes in the EPDS and MIBS scores throughout the COVID-19 pandemic. The increase in daily reported number of confirmed COVID-19 patients affected the psychological distress of the postpartum mothers and also caused social restrictions. A previous study in Wuhan showed that the prevalence of perinatal depression increased more as the COVID-19 pandemic worsened more [55]. However, our results showed that there was no changes of the average sum scores of the EPDS and MIBS. The third wave of COVID-19 cases has arrived in Japan since December 2020, and a state of emergency was issued again in January 2021 [38]. Because of the effects of chronic stress and the state of emergency, it is essential to observe future trends.

### Community-based integrated care system and implementation

Possible barriers to the use of medical services must be considered. Middle-aged adults, women, and those who have experienced panic symptoms reportedly use mental health services less frequently after disasters [39]. In a UK study, women who died by suicide during the perinatal period were less likely to have contacted any psychiatric services before their suicide, compared with non-perinatal women [56]. In addition, because of the COVID-19 pandemic, mothers are being forced to restrict the use of medical services. During the COVID-19 pandemic, pregnant and postpartum women have experienced the cancellation of planned social support, and young people who had mental health problems were not able to access mental health support [30, 57].

Perceived supports provided by health care staff can be regarded as protective factors against stress-related symptoms [21]. Besides, a community-based integrated approach, including suicide prevention, may contribute to the maintenance of peripartum mothers’ mental health [58, 59]. Our results suggest the need to strengthen both general public health interventions and mental health care services during the COVID-19 pandemic. Suicide prevention for perinatal women is also important. Online support may be an optimal and effective option to assess each mother’s psychological and social needs, and such support might reduce psychological distress and prevent adverse effects on long-term mental health [29, 58, 60, 61].

Previous research has shown that an important factor associated with postpartum depression during the COVID-19 pandemic was immigration status [18]. Foreign nationals are unevenly distributed in metropolitan areas in Japan, and foreign nationals account for about 4 % of the population in eastern Kanagawa [62]. We revealed that foreign nationals are less likely to contact appropriate services for mental illness; therefore, a community-based integrated care system that is accessible to foreign nationals during the perinatal period is also needed [63]. Further research is required to clarify individual responses and resilience, taking into account social, economic and cultural contexts.

### Strengths and limitations

Many of the studies that are currently being published have been conducted using online surveys because of convenience and COVID-19 precautions [6, 21, 30]. Online surveys inevitably encounter the problem of spoofing, which is detrimental to an accurate understanding of mental health. On the other hand, the screening questionnaire used in our study was completed face-to-face at the hospital by midwives. Furthermore, although a small number of missing data were excluded, this was a retrospective study of almost all the mothers who gave birth at one regional core medical institution. As a result, our study had a reduced sampling bias and might be a valuable resource for understanding the actual state of perinatal mental health in a metropolitan area of Japan during the COVID-19 pandemic. Moreover, one strength of this study is that the impact of COVID-19 was clarified by comparing the scores obtained during the pandemic with those obtained in a control group that reflected the situation before the COVID-19 pandemic [30].

Some limitations of this study should be noted. First, the causal relationship between the present results and the COVID-19 pandemic could not be established because the study consisted of a retrospective chart review. Second, we relied on information derived from self-reported measures, and the participants consisted of mothers visiting a single hospital. Third, this study did not examine potentially confounding factors affecting postpartum mental health, such as educational level, current economic situation, social support, cultural context, and feelings toward pregnancy [18, 64]. Further studies to address these potentially confounding factors are needed.

## Conclusions

This study demonstrated the status of post-partum mental health during the COVID-19 pandemic, compared with the prior situation. Even in Japan where the number of newly confirmed COVID-19 cases is relatively small in the world, the COVID-19 pandemic has a negative impact on the mental health in postpartum mothers. Anxiety was significantly higher while anhedonia and depression were significantly lower during the COVID-19 pandemic, suggesting a state of hypervigilance. Our investigation may reflect COVID-19-related health concerns and the lack of social support caused by the COVID-19 pandemic. An optimal community-based integrated mental health care system for postpartum mothers is also required in the COVID-19 era. Further research is needed to clarify individual responses and resilience, taking into account social, economic and cultural contexts, and the long-term effects of the COVID-19 pandemic on postpartum mental health.

## Data Availability

The data sets used and /or analysed during the present study are available from the corresponding author upon reasonable request.

## Abbreviations

COVID-19: Coronavirus disease 2019
EPDS: Edinburgh Postnatal Depression Scale
MIBS: Mother-to-Infant Bonding Scale
STAI: State-Trait Anxiety Inventory
MANOVA: The multivariate analysis of variance
